# Natural Phytotherapeutics in Dermatology and Cosmetology: Bioactive Potential of Grape Pomace on Human Skin Fibroblasts

**DOI:** 10.3390/molecules30244679

**Published:** 2025-12-06

**Authors:** Barbara Domagała, Julia Orlińska, Małgorzata Duda, Zuzanna Setkowicz-Janeczko, Marzena Starzyk, Ewelina Piasna-Słupecka, Mariola Drozdowska, Ewa Godos

**Affiliations:** 1 Faculty of Biotechnology and Horticulture, University of Agriculture, 31-425 Krakow, Poland; 2 Department of Endocrinology, Institute of Zoology and Biomedical Research, Faculty of Biology, Jagiellonian University, 30-387 Krakow, Poland; 3 Laboratory of Experimental Neuropathology, Institute of Zoology and Biomedical Research, Faculty of Biology, Jagiellonian University, 30-387 Krakow, Poland; 4 Faculty of Food Technology, University of Agriculture, 30-149 Krakow, Poland

**Keywords:** grape pomace, *Vitis vinifera*, Regent, Rondo, Marechal Foch, flavonoids, antioxidants, fibroblasts, skin regeneration, biocosmetics, plant extracts

## Abstract

The aim of the study was to assess the biological potential of extracts obtained from the grape pomace of three *Vitis vinifera* hybrid varieties—Regent, Rondo and Marechal Foch—as a natural source of bioactive compounds, with possible application in cosmetology and dermatology. Grape pomace, which is an important by-product of the winemaking process, is a rich source of polyphenols, flavonoids, anthocyanins and vitamin C, which exhibit antioxidant, anti-inflammatory and cytoprotective properties that are important for skin health. The conducted studies determined the antioxidant activity of the extracts (DPPH) and the content of total phenolic compounds, flavonoids, anthocyanins and vitamin C, Of the varieties analysed: Marechal Foch exhibited the highest antioxidant activity (10 µmol TE/g), while Regent demonstrated the highest flavonoid content (50.42 mg/g) and vitamin C content (35.6 mg/100 g). The Rondo extract had the highest content of anthocyanins (362.36 mg/g) and total phenolic compounds (18.31 mg/g), indicating strong protective potential for skin cells. Regent extract at a concentration of 25 μg/mL was found to have the greatest effect on fibroblast proliferation and migration, significantly increasing the percentage of living cells and the rate of regeneration. This correlates with the high flavonoid content, which is particularly important for skin cells. These results confirm that grape pomace, particularly from the Regent variety, is a valuable source of natural antioxidants with anti-aging and regenerative properties. The use of these raw materials in cosmetic formulations aligns with the principles of the circular economy and the idea of “zero waste”, being an example of the sustainable use of by-products from the wine industry in the production of innovative bio-cosmetics.

## 1. Introduction

The production of wine generates significant amounts of waste, including grape pomace, accounting for around 20% of the weight of processed fruit—equivalent to up to 13 million tonnes per year worldwide [1,2,3]. Although this material is often treated as waste or fertiliser, it is actually a valuable source of biologically active substances with significant economic and health benefits. Pomace consists mainly of grape seeds, skins and stems, which are rich in fibre, polyphenols and other antioxidants [4].

Recent studies have consistently reported that proanthocyanidins are the main constituents of grape pomace, representing the largest fraction of extractable phenolics, followed by resveratrol and other stilbenes in smaller amounts. Their abundance is considered the key factor underlying the biological activities of grape pomace extracts, including antimicrobial, anti-inflammatory, and cytoprotective effects [3,5,6,7,8]. Grape skins are particularly rich in resveratrol with antioxidant, antiviral and preventive heart and skin disease properties, while the seeds contain proanthocyanidins that protect DNA from oxidative damage and promote tissue regeneration [9]. Resveratrol is extracted from grapes’ peel of the common red grape wine and exists in two different isoforms: the trans and the cis-resveratrol [10,11]

High-performance liquid chromatography (HPLC) is one of the most commonly used analytical techniques determining polyphenol content in plant materials and grape products. This method allows for the precise identification and quantification of both simple polyphenols, such as resveratrol, and more complex glycosides, such as verbascoside. Verbascoside and resveratrol were selected for quantification due to their well-established health-promoting properties, particularly their potent antioxidant and anti-inflammatory activities. High-Performance Liquid Chromatography (HPLC) with diode array detection (DAD) was employed as a standard, highly selective method for their simultaneous and accurate determination within the complex grape matrix [12,13,14].

In cosmetology and dermatology, grape pomace extracts are a highly valued plant raw material used in the production of bio-cosmetics. Thanks to their high polyphenol content, they show the ability to neutralize free radicals, protect the skin from oxidative stress, and improve its elasticity and hydration [3,6,7]. The flavonoids and anthocyanins they contain protect fibroblasts, which are cells responsible for synthesising collagen, elastin, hyaluronic acid and fibronectin—all of which are crucial for maintaining the structural integrity of the skin [15,16].

Human dermal fibroblasts are therefore a cellular model system for assessing the cytotoxicity and pro-regenerative activity of plant extracts used in cosmetology [17,18]. Such studies enable the safety and potential biostimulating effect of active substances of natural origin to be assessed.

Flavonoids, selected from a large group of phenolic compounds, are the main subject of this analysis due to their well-documented effect on skin condition. They exhibit antioxidant, anti-inflammatory, photoprotective, and fibroblast-stimulating proliferation effects by activating signaling pathways related to collagen renewal and inhibition of extracellular matrix metalloproteinases (MMPs) [3,8].

The growth of the winemaking industry in Central and Eastern Europe, coupled with the introduction of climate-resistant grape varieties such as Regent, Rondo and Marechal Foch, has expanded the potential applications of regional raw materials to include the cosmetics and pharmaceutical industries.

In this context, research into the biological activity of extracts from indigenous grape varieties aligns with the concepts of sustainable development and the circular economy (zero waste), making full use of by-products from the food industry.

**Figure 1:**
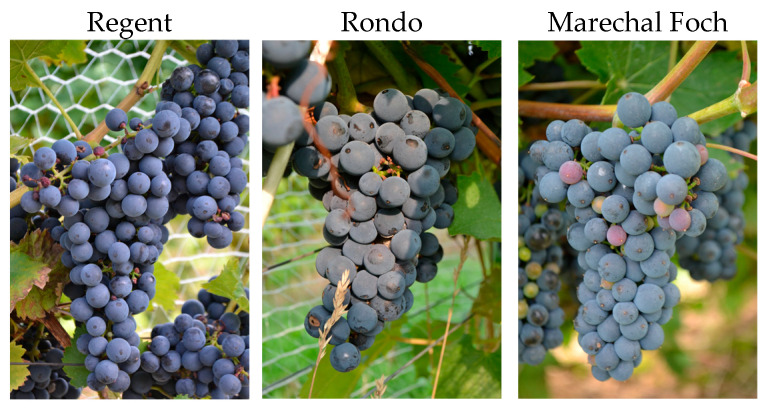
Grape clusters of the Regent, Rondo, and Marechal Foch cultivars. Photographs provided by the University Vineyard, University of Agriculture in Krakow.

The aim of this study was to assess the biological activity of extracts from the pomace of three grape varieties grown in Poland: Regent, Rondo and Marechal Foch (see Figure 1). The study also aimed to select the most promising variety as a raw material for cosmetics production, paying particular attention to their flavonoid content and their impact on the viability and proliferation of human fibroblasts in vitro. Specifically, the research aimed to determine the content of selected groups of phenolic compounds (flavonoids, anthocyanins, vitamin C, total phenols), analyse the relationship between the chemical composition of the extracts and their antioxidant activity, asses the impact of extracts on the survival and migration of human fibroblasts, select the raw material with the greatest cosmetic and regenerative potential, and assess the possibility of using wine pomace as an ingredient of biocosmetics in accordance with sustainable production principles.

## 2. Results

The analysis revealed significant variations in the chemical composition and biological activity of the tested grape varieties was found (see Table 1). Marechal Foch extract exhibited the highest antioxidant activity, indicating a significant ability to neutralise free radicals; however, this did not directly correlate with the content of phenolic compounds. The Regent variety had the highest flavonoid and vitamin C content, giving it strong reducing properties and supporting cellular protection mechanisms. However, the Rondo variety showed the greatest potential for further research due to its high anthocyanin and phenolic compound content. The high levels of these secondary metabolites, particularly anthocyanins, which have anti-inflammatory and antioxidant properties, suggest that Rondo extracts may offer the greatest protection to skin cells. This is why the next stage of the research focused analysing the flavonoid fraction of this variety, as this is a key factor in evaluating the dermatological and cosmetological potential of the obtained extracts. The results obtained were presented as mean values ± SD, with data subjected to analysis of variance (ANOVA) with Tukey’s post hoc test to determine significant differences between varieties (*p* < 0.05).

### 2.1. Chromatographic Analysis

Quantitative analysis of phenolic compounds revealed distinct differences among the tested grape varieties and sample types (Table 2 and Table 3, Supplementary Materials). The verbascoside content was significantly higher in lyophilised whole grape than in isolated skins. The highest concentration was observed in the Rondo variety (2.3381 mg/g DM), followed by the Regent (1.1548 mg/g DM). The lowest concentration was found in the Marechal Foch (0.1900 mg/g DM). In contrast, resveratrol levels were relatively low across all samples, although they remained slightly higher in lyophilizates than in skins. The highest concentration was observed in the Regent variety (0.1285 mg/g DM). Grape skins exhibited significantly lower levels of both compounds, suggesting that most phenolic compounds are retained in the pulp and seed matrix rather than in the epidermal tissues. Rondo demonstrated the richest polyphenolic profile, indicating a potentially higher antioxidant capacity than the other analysed cultivars.

### 2.2. Cell Viability Analysis

**Figure 2:**
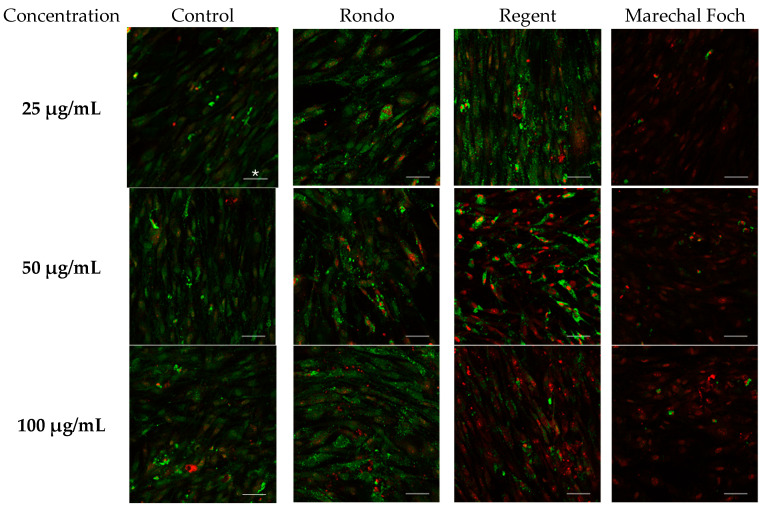
Results in the form of microphotographs from a confocal microscope of the LIVE/DEAD^®^ fibroblast viability study under the influence of 3 concentrations of extracts from the Rondo, Regent and Marechal Foch cultivars. * Each scale bar represents 100 nm.

Microphotographs showing the results of LIVE/DEAD^®^ staining revealed increased number of living cells emitting a green fluorescence signal from calcein was observed in all fibroblast cultures treated with the investigated extracts compared to the control group. The most pronounced protective effect was observed with extracts from the Rondo cultivar, particularly at concentrations of 25 μg/mL and 50 μg/mL, where an intense green signal dominated with only a small number of dead cells emitting a red EthD-1 signal. This demonstrates the highest cytoprotective potential of this variety extracts skin cells. In the case of Regent and Marechal Foch, however, this effect was less pronounced. At the highest concentration (100 μg/mL), a decrease in cell viability was observed. These results indicate that the Rondo extract most effectively supports the survival of fibroblasts and has a protective effect, which correlates with its high flavonoid and phenolic compound content. Microphotographs obtained using a confocal microscope are shown in Figure 2.

Quantitative analysis (presented in Table 4) was performed on fluorescence images obtained from three independent experiments (*n* = 3), each containing five microscopic fields per treatment group. Data are presented as mean ± SD, and statistically significant differences were determined using one-way ANOVA. Based on the results of the quantitative analysis, the lowest dose of extracts used was selected for further experiments.

Based on the results obtained, it can be concluded that all of the tested grape extracts demonstrate good cellular tolerance at lower concentrations. The Regent is safe for fibroblasts at a concentration of 25 μg/mL, with only 20% of cells dying, which indicates its potential biocompatibility. Increasing the concentration to 50 μg/mL did not cause a statistically significant decrease in cell viability; however, a clear decrease in the number of living cells was observed at 100 μg/mL.

A similar relationship was observed for the Rondo variety, for which a twofold increase in concentration from 25 to 50 μg/mL did not significantly impact the increase in the number of dead cells. In the case of the Marechal Foch extract, the results showed even a slight reduction in the percentage of dead cells at 50 μg/mL compared to 25 μg/mL, confirming the absence of significant changes in cytotoxicity.

All of the tested grape extracts at concentrations of up to 50 μg/mL can be considered safe for human fibroblasts. A clear cytotoxic effect only appears at a concentration of 100 μg/mL. These results suggest that the studied extracts, particularly those from the Regent variety, could be used s as protective ingredients in cosmetic and dermatological formulations.

### 2.3. Scratch Test

**Figure 3:**
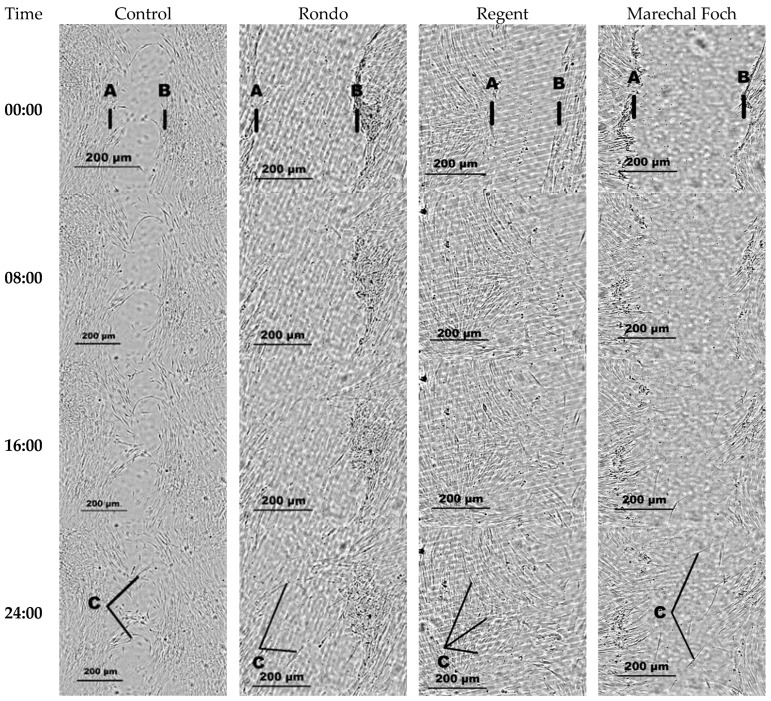
Microscopic images taken with the CytoSMART Lux2^®^ device for each sample applied and the evolution of wound healing in vitro as a function of time. A, B: denote the boundaries of the “wound”; C: fibroblast cells.

To investigate the effect of the extract added to the medium on fibroblast migration, the rate at which the migrating cells colonised the ‘wound’ (the cell-free area) was analysed. Using the CytoSMART™ Lux2 Live Cell Imager (CytoSMART Technologies B.V. Eindhoven, The Netherlands), time-lapse images were taken at 15 min intervals, and the area of the ‘wound’ was measured (in μm^2^). The results of a control group, in which the fibroblast culture did not contain extracts, were compared with those of a study group, in which the medium was enriched with plant extracts at a concentration of 25 μg/mL (Figure 3). The analysis was based on data obtained by measuring the wound area using imaging software (CytoSMART Connect 3.0).

The rate at which cells migrate to and colonise the surface (“wound”) is faster in the presence of the extract from the Regent variety pomace (this differs statistically significantly from the rate at which cells of the control group settle on the surface). However, statistical analysis did not reveal any significant differences in wound healing rates in the presence of extracts from the other two grape varieties.

A nonparametric Spearman’s rank test was used to analyse the results of the scratch test (grape variety). This method made it possible both to assess significant differences between groups and analyse the direction and strength of the relationship in the “wound healing test”.

The most structured and effective wound healing process is observed in the case of the Regent variety. Not only do fibroblasts in this condition close the fissure quickly, achieving full wound closure within 24 h, they also maintain a highly directional orientation consistent with the substrate during migration. This indicates that the addition of Regent extract promotes orderly and directed cell movement, a desirable feature in biomedical engineering tissue regeneration processes where precise cell reorientation is essential for restoring structural functionality. Unlike the other two *V. vinifera* varieties, wound closure is achieved via an orderly migration front rather than clear cell aggregation/clusters.

## 3. Discussion

Comparing our data with the literature, it should be emphasized that the concentrations of the extracts used differed depending on how the raw material was prepared. Nizioł-Łukaszewska [19] demonstrated that water-glycerin and glycol extracts stimulate fibroblast proliferation at higher concentrations (6.25–100 mg/mL), whereas in our study the beneficial effect was observed at much lower concentrations (25 μg/mL). These differences can be explained by the type of plant material used and extraction method. The presence of solvents also significantly impacts the results—as indicated in the work of Wyganowska-Świątkowska [20], high concentrations of ethanol can be toxic to fibroblasts, a factor that should be considered in future studies.

Based on the results obtained, it can be concluded that the greatest increase in fibroblast viability occurred when the Regent pomace extract was added to the culture medium at the lowest concentration (25 μg/mL). This effect is likely due to the high flavonoid content (0.5042 mg QE/100 g) and vitamin C content (35.6 mg/100 g FW). The main flavonoids present in wine pomace—quercetin and catechin—exhibit strong antioxidant and anti-inflammatory properties, which may have increased fibroblast viability. Vitamin C, on the other hand, is known for its key role in collagen synthesis and cell growth under oxidative stress and is probably contributed to the accelerating cell migration in the wound healing assay [21,22].

The observed protective and regenerative effects of grape pomace extracts on human skin fibroblasts can be attributed to their high polyphenolic content and associated antioxidant capacity. Polyphenols such as catechins, proanthocyanidins, and resveratrol inhibit the activity of collagenase and elastase, thus protecting extracellular matrix components from degradation and contributing to skin firmness and elasticity [23]. In addition, grape seed extract has been shown to protect human dermal fibroblasts against UVA-induced oxidative stress, improving their viability and supporting collagen stability [24]. Resveratrol, a major grape-derived polyphenol, exhibits antioxidant and anti-inflammatory activity that promotes wound healing by stimulating fibroblast proliferation, migration, and extracellular matrix deposition while suppressing ROS accumulation [25]. These effects may, at least in part, involve modulation of the sirtuin signaling pathway. Recent studies emphasize the pivotal role of SIRT1 in maintaining fibroblast redox homeostasis, DNA repair, and cellular longevity, while its decline contributes to both intrinsic and photoaging processes [26,27]. Although our study focused primarily on protective effects in unstressed fibroblasts, further investigation using oxidative stress-induced models is warranted. Hydrogen peroxide (H_2_O_2_)-induced premature senescence (SIPS) in fibroblasts is a well-established in vitro model that mimics oxidative damage and allows testing of potential anti-aging compounds [28]. Such an approach could provide deeper insight into the mechanisms through which grape pomace extracts restore redox balance and cellular repair capacity. Furthermore, proanthocyanidins have been demonstrated to protect dermal fibroblasts by maintaining mitochondrial function and enhancing collagen biosynthesis, reinforcing their role as promising natural anti-aging agents [29].

Skorek’s [30] observations, which showed that plant extracts stimulate fibroblast proliferation in vitro at concentrations below 1 mg/mL and have an inhibitory effect at concentrations above 2.5 mg/mL. The differences in the values obtained may be due to the way the samples were prepared—in this study, the extraction was carried out using freeze-dried pomace (skins and seeds), whereas Skorek only tested extracts from grape skins. According to Negro [31], grape seeds contain significantly more phenolic compounds than skins, justifying the need for lower concentrations of extracts obtained from whole pomace. Similar conclusions were presented by Melo [21], who evaluated the cytotoxicity of grape pomace extracts against mouse macrophages RAW 264.7. The authors showed that concentrations below 10 μg/mL exhibited low cytotoxicity, whereas higher concentrations (50–100 μg/mL) significantly reduced cell survival. These results partially coincide with the results of this study, in which a concentration of 25 μg/mL was considered safe. The differences may be due to both the different cell models and the grape varieties used. Maluf [32], in turn, confirmed the high antioxidant activity of *V. labrusca* pomace, demonstrating its ability to protect 3T3 fibroblasts from H_2_O_2_-induced oxidative stress. This suggests the potential for its use as a natural antioxidant in cosmetics. Additionally, Kabir [33] showed that grape seed extract effectively protects MA104 cells from AAPH-induced oxidative stress. Letsiou [34] also demonstrated that *V. vinifera* leaf extract increases the viability of human fibroblasts and activates the expression of the SIRT1 and HSP47 genes, which are responsible for antioxidant and anti-aging processes. This confirms the potential of grapevine as a source of bioactive compounds for cosmetic use.

The metabolite profile of wines is shaped primarily by cultivar, vintage, and environmental origin [35,36]. The grape genotype strongly determines the composition of key metabolite classes, particularly polyphenols, anthocyanins, and triterpenoids, with disease-tolerant varieties often showing markedly higher anthocyanin levels and distinct diglucoside profiles compared with traditional *Vitis vinifera* [37]. Vintage exerts an even stronger influence on overall wine chemistry, as demonstrated by clear PCA-based separation of wines from different years and substantial year-to-year variability in anthocyanins, resveratrols, sulfur volatiles, and basic parameters such as alcohol content [36,38,39]. Environmental conditions, including soil and climate, further modulate phenolic composition—especially resveratrol levels—and contribute to characteristic terroir-dependent metabolic fingerprints [39]. Although some studies show limited discrimination by geographical origin alone, differences in specific metabolites (e.g., organic acids, minerals, or certain aroma compounds) can still reflect local growing conditions [37]. Vineyard practices and plant health additionally affect the biosynthesis of phenolics and the relative proportions of anthocyanin families [35,36]. Overall, vintage is the dominant factor differentiating the global chemical profile of wines, while cultivar determines the metabolic potential of the plant, and environmental conditions fine-tune the final composition [37].

Analysis of polyphenol content in whole fruit lyophilisates and in isolated grape skins showed significant differences in the distribution of verbascoside and resveratrol depending on tissue fraction and variety. While initial HPLC analyses utilized freeze-dried pomace, the current study focused on fresh grape skins to verify previous chemical trends, necessitated by constraints of the annual wine production cycle. As indicated in Table 2, concentrations of both compounds were significantly higher in lyophilizates (which consisted of skins, seeds and stems), compared to skins samples, which indicates that secondary metabolites, including stilbenes (resveratrol) and hydroxycinnamonates (verbascoside) accumulate in both the skins and seeds [40]. In addition, verbascoside was the dominant compound in all samples, suggesting that it is a key phenolic metabolite in the analysed cultivars.

Resveratrol, as a representative of stilbenes, has been repeatedly determined using HPLC in various biological matrices. Moyano-Méndez et al. [41] described the use of HPLC to monitor the stability of trans-resveratrol and its isomerization to the cis form. Verbascoside, as a polyphenolic glycoside, can also be determined by HPLC. The literature emphasizes that the determination of its content in plant extracts requires the application of appropriate separation conditions. The analysis of polyphenols from grapes and by-products of winemaking [42,43] indicates that such mobile systems are most efficient for the separation of both simple phenols and their glycosides. According to research conducted by Rockenbach [44], red grape extract (Cabernet Sauvignon variety) provided 0.0402 mg/g dry weight of resveratrol. In the study by Boonchu [45], the value obtained was 0.0049 mg/g DM (Black queen variety). In our samples, however, the values ranged between 0.034 and 0.175 mg/g DM, depending on the variety. In the context of health value research, the highest total concentration of polyphenols in the skins of the ‘Rondo’ cultivar (2.3381 mg/g DM) highlights the potential of this material as a source of valuable bioactive compounds, surpassing ‘Regent’ and ‘Marchal Foch’ in this respect. However, although total phenolic values were generally consistent with those reported by other authors, it is well known that they are closely related to grape variety, geographical origin, winemaking practices, and extraction methodology [46,47,48,49].

The results of this study suggest that grape pomace extracts from the Regent variety, particularly at low concentrations, can promote fibroblast viability and support wound healing processes through the synergistic action of flavonoids, anthocyanins and vitamin C. Further research should focus on standardizing the composition of the extracts and investigating the molecular mechanisms underlying their cytoprotective and regenerative properties. Further analysis of the chemical composition is worth carrying out, including determining the iridoid glycoside content using high-performance liquid chromatography. Future studies, should also evaluate the expression of proteins that regulate proliferation and regenerative processes in fibroblasts. We acknowledge that the current study focused primarily on protective effects in non-stressed fibroblast cultures. Future experiments will employ a hydrogen peroxide (H_2_O_2_)-induced oxidative stress model to evaluate the regenerative and repair potential of grape pomace extracts under conditions that better mimic skin damage and oxidative imbalance. This approach will provide a more comprehensive insight into the mechanisms responsible for cellular recovery.

The conducted studies confirmed the high biological potential of grape pomace extracts as a valuable source of natural bioactive that protect and regenerate skin cells. The results indicate that the phenolic compounds, flavonoids and anthocyanins found in the extracts, particularly those from varieties cultivated in Central and Eastern Europe, could be effective ingredients in anti-ageing and cytoprotective cosmetic and dermatological products. These results are an important step towards developing new, sustainable plant-based materials that combine biological efficacy with ecological considerations and the principles of the circular economy in the cosmetics industry.

Two methodological limitations should be noted. Firstly, anthocyanin concentrations were calculated using the molar extinction coefficient of cyanidin-3-glucoside rather than a calibration curve. While this method is widely used for comparative data, establishing a calibration curve with cyanidin-3-glucoside in the same solvent system will be implemented in future work to improve quantitative accuracy and allow for determination of linearity, LOD, and LOQ. Secondly, compound concentrations were primarily normalized to dry weight, and though extraction yields were within the expected range (22.8–30.1%), this variability may influence absolute quantitative comparisons between cultivars.

## 4. Materials and Methods

### 4.1. Plant Material

In September 2023, 2 kg of ripe fruits were taken from the university vineyard of the University of Agriculture in Cracow in Garlica Murowana (50°08′22″ N 19°55′38″ E) from three red grape varieties of the type: Rondo, Regent and Marechal Foch. Juice was squeezed out of each variety separately and the pomace was separated, which was then frozen at −18 °C. After 2 weeks, half of the frozen pomace was freeze-dried, while the other half of the frozen samples were chemically analyzed. For comparative analyses, in September 2025, a separate batch of ripe fruits (approx. 1 kg per variety) from the same vineyard was collected. Fresh grape skins were immediately isolated after destemming and pressing, with a 10 g subsample taken from each variety.

Extraction yield was determined gravimetrically (relative to dry material) to enhance comparability between cultivars. The ethanol/HCl extraction applied in this study resulted in yields of 27.4% for Regent, 30.1% for Rondo, and 22.8% for Marechal Foch. These values fall within the ranges reported for similar ethanolic extractions of red grape pomace rich in phenolics (typically 18–32%) [50,51] and 12–25% for isolated grape skins [52,53]. All phytochemical determinations were expressed on a dry-weight basis; however, extraction yield values are provided to increase the transparency of the experimental procedure. Initial pomace samples were dried at 65 °C, but recognizing the potential for thermal degradation of thermosensitive polyphenols, particularly anthocyanins [54,55,56], subsequent grape skin samples were dried at 30 °C. Anthocyanin quantification was performed spectrophotometrically at 520 nm using the molar extinction coefficient of cyanidin-3-glucoside. This quantification method is supported by the literature for use in comparative analyses when certified standards are absent [55].

### 4.2. Vitamin C

The test was carried out using a modified iodometric method involving titration of samples with a solution of iodine in potassium iodide, using starch as an indicator. Titration was carried out until a dark blue-violet color was achieved. The test was carried out as follows: 12.5 g of pomace was added to a blender and ground with 50 mL of 1% oxalic acid (Stanlab, Lublin, Poland), then drained. The filtered material was placed in 5 mL beakers and 1 mL of 1% starch solution (5 g/L plus 200 NaCl, Chempur, Piekary Śląskie, Poland, Eurochem; Tarnow, Poland) was added. The next stage of the test was titration with a potassium iodide iodine solution (1:1 ratio, Fluka, POCH, Gliwice, Poland) until a blue color persisting for 30 s was obtained. Then, the iodine solution in potassium iodide was titrated using 0.1% ascorbic acid (0.0250 g/25 mL of distilled water, POCH, Gliwice, Poland) with three repetitions of 1 mL of solution with 1 mL of starch. The results obtained were converted to ascorbic acid content in mg/100 g.

### 4.3. Total Phenolic Compounds

This test was performed according to the Folin-Ciocalteau method as follows: 2 g of pomace was measured into a mortar, which was then ground with 10 mL of 80% methanol (Stanlab, Lublin, Poland). For the next 10 min, the ground material was centrifuged at 6000 rpm at a temperature of 18 °C. In the next stage, 2 mL of Na_2_CO_3_ (2%, Eurochem, Tarnow, Poland) was added, left for 5 min, and 0.1 mL of Folin-Ciocalteau reagent solution (in a 1:1 ratio with distilled water, Chempur, Piekary Śląskie, Poland) was added. Phenolic compounds undergo oxidation when in contact with phosphomolybdenum and phosphotungstic acid salts contained in the Folin reagent. During this reduction, their color changes to blue. At the same time, a blank sample was prepared, consisting of 0.1 mL of 80% methyl alcohol, 2 mL of 2% Na_2_CO_3_, and 0.1 mL of Folin reagent solution. The prepared samples were mixed and placed in the dark for 45 min. After this time, the absorbance of all samples was measured in a Helios Beta UV-VIS spectrophotometer (Thermo Electron Corporation, Vendenheim, France) at a wavelength of 750 nm. A calibration curve was prepared based on standard solutions of gallic acid (anhydrous, Merck, Darmstadt, Germany) in the range of 0–20 mg/L. The results were substituted into the absorbance curve formula, based on which the results were obtained in mg/mL, which were then converted to mg/g.

### 4.4. Anthocyanin

To measure the anthocyanin content, the method developed by Lee [54] was used. For this purpose, 0.5 g of pomace was ground in a mortar with 10 mL of 96% ethyl alcohol (Stanlab, Lublin, Poland) and 1 mL of hydrochloric acid (35–38%, Stanlab, Lublin, Poland) mixed in a ratio of 85:15. The resulting extract was centrifuged at 18 °C for 10 min at 6000 rpm. The centrifuged extract was then diluted 10 times and its absorbance measured at a wavelength of 520 nm in a Helios-Beta UV-VIS spectrophotometer (Thermo Electron Corporation, Vendenheim, France). The anthocyanin content was calculated based on the molar extinction coefficient and the molar mass of cyanidin-3-glucoside. No calibration curve was performed. The anthocyanin content was given as cyanidin-3-glucoside equivalent [mg/g].

Anthocyanin content was calculated using the molar extinction coefficient and molecular weight of cyanidin-3-glucoside. This spectrophotometric approach is commonly applied when quantitative standards are not available and provides reproducible comparative data across samples measured under identical conditions. However, we acknowledge that the absence of a dedicated calibration curve in the same solvent system represents a methodological limitation, meaning the obtained values should be interpreted as estimates based on the molar absorptivity of cyanidin-3-glucoside.

### 4.5. Flavonoid Content

This study used the reaction of flavonoids with AlCl_3_, which undergo staining in an acidic environment. Flavonoids were determined in terms of quercitin. For the purpose of the test, 2 g of plant material was rubbed in a mortar with 10 mL of methanol at a concentration of 80% (Stanlab, Lublin, Poland). Then it was centrifuged at 18 °C for 10 min at 6000 rpm. The centrifuged samples were diluted ten times with distilled water and 1 mL was taken from it. 0.1 mL of AlCl_3_ (Chempur, Piekary Śląskie, Poland) and 1.4 mL of methanol acidified with 5% acetic acid (POCH, Gliwice, Poland) were added to each of the tubes. In blank trials, 1.5 mL of acidified methanol was added to 1 mL of material. In the zero test, 1 mL of AlCl_3_ solution and 1.4 mL of acetic acid-acidified methanol were added to 1 mL of 80% methyl alcohol. All samples were placed in the dark for 30 min. After this time, the absorbance of the test and blank samples was measured in the UV-VIS Helios Beta spectrophotometer (Thermo Electron Corporation, Vendenheim, France) at a wavelength of 415 nm. The difference between the absorbances of the two samples was calculated and the differences were substituted for the curve formula. The results obtained in mg/mL were converted to mg/g.

### 4.6. Chromatographic Analysis of Plant Extracts (HPLC-DAD Method)

Chromatographic analysis was performed using an Agilent 1100 HPLC system (Agilent Technologies, Waldbronn, Germany) equipped with a DAD G1315 diode-array detector (Agilent Technologies, Santa Clara, CA, USA). Separation was carried out on a Thermo Scientific ODS Hypersil column (250 × 4.6 mm, 5 µm) (Thermo Scientific, Waltham, MA, USA) at room temperature. The injection volume was 20 µL, and the flow rate of the mobile phase was maintained at 1.0 mL/min. The mobile phases consisted of A—acetonitrile, B—0.1% formic acid solution, and C—methanol. The elution program included an isocratic phase from 0 to 9 min (0.6% A and 99.4% B), followed by a linear gradient from 10 to 80 min reaching 0.6% A, 9.4% B, and 90% C, and finally returning to the initial conditions (0.6% A and 99.4% B) between 80 and 85 min. Detection was performed at 210 nm and 330 nm. Sample preparation involved weighing 200 mg of the dried plant material and extracting it three times with 2 mL of methanol acidified with 0.1% hydrochloric acid. The combined extracts were filtered through a 0.45 µm membrane filter prior to HPLC analysis. The applied chromatographic method demonstrated excellent reproducibility and signal stability. Retention time deviations did not exceed 2%, while the relative standard deviation (RSD) for the main peak areas was below 3%. The method ensured sufficient resolution of bioactive compounds and proved suitable for the analysis of complex plant extracts with diverse chemical profiles.

The quantification data for verbascoside and resveratrol content, presented as the mean +/− standard deviation (SD) were statistically evaluated to ascertain significant differences across the grape varieties (Regent, Rondo and Marechal Foch) and the tissue types (lyophilizates and grape skins). The simultaneous influence of both factors, variety and tissue type, on polyphenol content was assessed using a two-way analysis of variance (ANOVA). Following the identification of significant main effects or interactions by ANOVA, mean separation was achieved using Tukey’s Honest Significant Difference (HSD) post hoc test to determine which specific groups differed from one another. Differences were considered statistically significant at a probability level of *p* < 0.05. Lowercase letters or symbols superscripted in Table 2 denote statistically significant differences (*p* < 0.05) between the compared means.

While the primary supporting analyses utilized lyophilized pomace from the previous season, the current study employed fresh grape skins to verify observed chemical trends, a necessitated modification due to the constraints of the annual production schedule.

### 4.7. Antioxidant Activity

The study of antioxidant activity was investigated by colorimetric method based on the reduction of the DPPH radical (2,2 diphenyl-1-picrylhydrazyl). This solution, under the influence of antioxidants present in the pomace, changes color from purple to yellow. To carry out the test, 2 g of plant material was weighed, which was ground in a mortar with sand and 10 mL of 80% methyl alcohol (Stanlab, Lublin, Poland), and then centrifuged at 18 °C at 6000 rpm. 1 mL of perfiltrate and 4.9 mL of DPPH solution (Aldrich, Steinheim, Germany) were added to the previously prepared test tubes. During this time, a comparator solution consisting of 0.1 mL of methanol and 4.9 mL of DPPH solution was also prepared. All samples were thoroughly mixed and placed in the dark for 15 min. After this time, the absorbance of the analytical samples and the comparator solution was determined in the UV-VIS Helios Beta spectrophotometer (Thermo Electron Corporation, Vendenheim, France) at a wavelength of 517 nm. The apparatus was zeroed with 80% methyl alcohol.

To ensure comparability and provide a standard reference, Trolox (6-hydroxy-2,5,7,8-tetramethylchroman-2-carboxylic acid, 99.9%, Merck, Darmstadt, Germany) was utilized as the antioxidant reference standard. A calibration curve was prepared using Trolox standard solutions in 80% methanol across a range of 0 to 50 µmol/L. The radical scavenging activity of the Trolox standards was measured identically to the samples. The calibration curve established the relationship between the percentage of radical scavenging and the concentration of Trolox (with R^2^ > 0.99). The results were subsequently expressed as the Trolox Equivalent Antioxidant Capacity (TEAC), calculated from the linear equation of the Trolox standard curve. Final antioxidant activity is presented in the standard units of µmol TE per gram of dry material (µmol/TE/g), as recommended for comparative studies.

### 4.8. Preparation of Plant Extracts

The extraction was carried out based on the procedure described by Alsaraf [55], with minor modifications adapted to the test material. This method was chosen because of its documented effectiveness in isolating biologically active compounds from plant raw materials. 25 g of freeze-dried plant material was ground into a fine powder using a stainless steel grinder, then extracted in 70% ethanol (20 mL) at 37 °C overnight. The fraction extracted with ethanol was separated by gauze and filtered through filter paper. The filtered extract was then concentrated in a rotary evaporator. Working concentrations were prepared from the evaporated extract. In the studies, plant extracts were added to the cultured cells in concentrations: 25 μg/mL, 50 μg/mL and 100 μg/mL. After a preliminary analysis of fibroblast viability, a concentration of 25 μg/mL was selected for further analysis. To exclude any solvent-related effects, a vehicle control group was included in all experiments. Fibroblasts were treated with 0.1% (*v*/*v*) ethanol in culture medium, corresponding to the highest concentration of solvent used to dissolve grape pomace extracts. No cytotoxic effect of ethanol at this concentration was observed. Each experimental condition was performed in triplicate (*n* = 3) using independent biological replicates. Statistical analysis was conducted using one-way ANOVA followed by Tukey’s post hoc test (GraphPad Prism 9.0), with statistical significance set at *p* < 0.05.

### 4.9. Preparation of Fibroblasts

A stable line of human skin fibroblasts has been made available for experiments by the Department of Cell Biology of the Faculty of Biochemistry, Biophysics and Biotechnology at the Jagiellonian University. The cells were isolated from skin fragments (biopsy) taken from three healthy donors (two women aged 32 and 67 and one man aged 48) during plastic surgery. The study was approved by the Ethics Committee of the Jagiellonian University in Krakow, Poland (Approval number: KBET/480/B/2003). Each 0.5 cm^2^ skin sample was washed with a buffered saline solution (PBS; buffered saline, without calcium and magnesium ions), supplemented with an antibiotic mixture consisting of 5000 U/mL penicillin and 5 mg/mL streptomycin (P/S; all from Sigma-Aldrich, Steinheim, Germany). The rinsed skin was cleaned of fat and blood vessels, and then incubated in a solution of the enzyme—diapase (6 U/mL in PBS; Gibco, NY, USA) at 4 °C for 16 h, for initial enzymatic dissociation. Fibroblasts were isolated using collagenase I solution (1 mg/mL in DMEM, Sigma-Aldrich, Steinheim, Germany) culture medium at 37 °C for 16 h. The cells obtained in this way were cultured in DMEM medium with the addition of 10% bovine serum (FBS) and with the addition of P/S, in an incubator under constant conditions: 37 °C, 5% CO_2_ in air, maximum relative humidity [56]. After reaching 80% confluence, the cells were flushed with PBS solution and suspended in trypsin and incubated at 37 °C until the cells detached from the surface. After detaching the cells, to inactivate trypsin, 9 mL of fresh culture medium was added to the cells. Then some of the cells were used to conduct experiments, and the rest were returned to culture [41,57].

Cells for the LIVE/DEAD^®^ fluorescence analysis and wound healing assay (“scratch test”) tests were seeded on 8-chamber culture slides (Lab-TekTM II—CC2, poly-L-lysine; Nunc™). Their density was 6 × 10^4^ cells/chamber.

### 4.10. Cell Viability Analysis

The LIVE/DEAD^®^ Viability/Cytotoxicity Kit (Invitrogen™, Thermo Fisher, Waltham, MA, USA) was used to perform cell viability analysis, which allows staining living and dead cells. The kit contains two dyes: calcein, which, under the influence of esterase activity, an enzyme present in the cell membrane, is converted from a non-fluorescent form to a form that gives green fluorescence, and ethidine homodimer (EthD-1), which penetrates cells with damaged membranes and as a result of binding to nucleic acids, gives a red fluorescence signal. All reagents used for fluorescent dyeing come from the manufacturer. The components of the putty (calcein and EthD-1) were thawed on ice and brought to room temperature.

The fibroblasts were seeded on chambers, then incubated for 24 h in a culture medium, after which the culture medium was replaced with a medium containing 25 μg/mL, 50 μg/mL and 100 μg/mL extracts from grape pomace of the Regent, Rondo and Marechal Foch varieties and incubated for another 7 days. The cells were flushed twice with warm PBS without Ca^2+^ and Mg^2+^ ions (37 °C). A 4 μM solution of EthD-1 was then prepared by dissolving an appropriate amount of 2 mM of the starting solution in PBS and mixed. An appropriate volume of calcein was added to 4 μM of EthD-1 solution so that its final concentration was 2 μM. The whole thing was mixed intensively. PBS was removed from the culture wells using a pipette, and 100 μL of the resulting mixture (calcein/EthD-1) was added to the cells for the test. The whole thing was incubated for 40 min at room temperature. After the incubation was completed, the cells were rinsed twice with PBS. The preparations were then sealed using VectaMount AQ gel (Biokom, Janki, Poland). The slides were imaged using the OLYMPUS FV1200 FLUOVIEW laser confocal microscope (OLYMPUS, Tokyo, Japan) at an excitation wavelength in the range of 494 nm for calcein and 528 nm for EthD-1 and a detection of 517 nm for calcein and 617 nm for EthD-1.

In order to compare the effect of selected extracts on the viability of human fibroblasts, a LIVE/DEAD^®^ staining kit was used to differentiate living and dead cells. The percentage of living cells was estimated based on random observation of 5 areas at low (10×) magnification. In each of the five areas, all cells in the field of vision that made up 100% of the total were counted, and then the number of living and dead cells was counted, respectively. An average was drawn from the observed 5 areas, and a statistical analysis was carried out.

### 4.11. Scratch Test

To analyse the rate of cell migration and wound healing, special Culture-Insert 4 Well in μ-Dish (IbiTreat 35 mm high) vessels were used. These vessels have 4 small chambers into which cells are inserted—removing the walls separating the chambers from each other creates the so-called wound. The cells were cultured in the presence of pomace at a concentration of 25 μg/mL for 7 days. A wound healing test was then performed, measuring the rate of wound healing over 24 h (0 h, 8 h, 16 h, 24 h) using the CytoSMART™ Lux2 Live Cell Imager (IVIM TECHNOLOGY, Daejeon, Republic of Korea) and software provided by the manufacturer.

## 5. Conclusions

1. Although Marechal Foch exhibited the highest antioxidant activity among the tested samples, its limited ability to promote the viability and migration of fibroblasts was comparable to the control group due to its relatively low content of flavonoids, anthocyanins and vitamin C.
2. Despite having the highest overall content of anthocyanins and phenolic compounds, Rondo did not demonstrate significantly higher fibroblast migratory activity. This may be due to its lower levels of flavonoids and vitamin C compared to Regent.
3. The content of the bioactive polyphenols, verbascoside and resveratrol, was observed to be significantly higher in the lyophilisates of the whole fruits compared to the corresponding peel fractions. This finding designates the whole-fruit lyophilisate as the most valuable fractional raw material for potential application in the nutraceutical industry. Specifically, the Rondo and Regent varieties exhibited the highest absolute content of these compounds.
4. The greatest stimulation of fibroblast viability and migration was observed after the application of Regent extract at a concentration of 25 μg/mL. This correlates with the extract’s high flavonoid and vitamin C content and its strong antioxidant activity.
5. The results suggest that the Regent variety shows a higher potential for cosmetic applications.
6. Wine pomace is a valuable source of natural antioxidants and can be used by the cosmetic industry to develop anti-aging and protective products.

## Figures and Tables

**Table 1:** Chemical composition of extracts from the Rondo, Regent and Marechal Foch cultivars.

Variety	Antioxidant Activity TEAC [µmol TE/g d.w. *]	Total Flavonoid Content Calculated as Quercetin Acid Equivalent [mg QE/g]	Vitamin C [mg/100 g f.w. **]	Total Phenolic Compounds Calculated as Gallic Acid [mg GA/g f.w.]	Anthocyanins Calculated as Cyanidin-3-glucoside [mg/g]
Regent	9.64 ± 0.08 ^b^	50.42 ± 2.81 ^a^	35.6 ± 1.8 ^a^	11.93 ± 0.74 ^b^	161.42 ± 9.33 ^b^
Rondo	9.73 ± 0.02 ^b^	32.81 ± 1.92 ^b^	27.4 ± 1.2 ^b^	18.31 ± 0.65 ^a^	362.37 ± 11.54 ^a^
Marechal Foch	10.00 ± 0.00 ^a^	17.31 ± 1.11 ^c^	21.9 ± 1.0 ^c^	13.98 ± 0.52 ^b^	59.56 ± 4.26 ^c^

The values with different superscripts differed significantly (*p* < 0.05). * d.w.—dry weight, ** f.w.—fresh weight.

**Table 2:** Content of verbascoside and resveratrol in samples of grapes lyophilizates and their skins.

	Lyophilizates—Variety			Grape Skins—Variety		
Polyphenols (mg/g DM)	Regent	Rondo	Marechal Foch	Regent	Rondo	Marechal Foch
Verbascoside	1.1548 ± 0.05 ^c,x^	2.3381 ± 0.08 ^a,y^	0.1900 ± 0.01 ^d,x^	0.0071 ± 0.00 ^d,x^	0.3646 ± 0.02 ^b,x^	0.0024 ± 0.00 ^d,x^
Resveratrol	0.1285 ± 0.01 ^b,x^	0.1754 ± 0.01 ^a,z^	0.0339 ± 0.00 ^c,z^	0.0731 ± 0.00 ^b,x^	0.0722 ± 0.01 ^b,y^	0.0324 ± 0.00 ^c,z^

Statistical differences markings: the letters (a,b,c,d) indicate the differences between the varieties in a given sample, and the symbols (x,y,z) indicate the differences between the samples in a given variety, *p* < 0.05.

**Table 3:** Validation parameters for HPLC-DAD method.

Parameter	Verbascoside	Resveratrol
**Calibration range (mg/mL)**	0.05–1.00	0.05–1.00
**Calibration equation**	y = 1218.16x − 14.89	y = 5245.60x − 75.66
**R^2^**	0.9983	0.9985
**Residual SD**	19.67	78.51
**LOD (mg/mL)**	0.053	0.049
**LOQ (mg/mL)**	0.161	0.150
**Repeatability (CV%)**	<2%	<2%

**Table 4:** Analysis of the viability of LIVE/DEAD^®^ fibroblasts after culture in the presence of Rondo, Regent and mf extracts. the results of the determination of the number of living/dead cells were given as mean ± sem, *n* = 3. The values with different superscripts differed significantly (*p* < 0.05, *t* test). Statistical differences are marked with letters (a–f) and symbols *, ** and ***.

	Rondo		Regent		Marechal Foch	
	% Live	% Dead	% Live	% Dead	% Live	% Dead
Control (0 μg/mL)	97.2 ± 1.5 ^f^	2.8 ± 0.6	96.5 ± 1.2 ^f^	3.5 ± 0.5	95.9 ± 1.8 ^f^	4.1 ± 0.8
25 μg/mL	79.4 ±2.1 ^a^	20.6 ± 1.1 **	86 ± 2.5 ^c^	14 ± 1.3 *	68 ± 2.1 ^e^	32 ± 7.1 ***
50 μg/mL	77.18 ± 3.4 ^a^	22.82 ± 1 **	77.8 ± 4.2 ^a^	22.21 ± 1.2 **	69.5 ± 3.4 ^e^	30.5 ± 5.1 ***
100 μg/mL	56.6 ± 6.1 ^b^	43.4 ± 6.1 ***	48.12 ± 5.1 ^d^	51.88 ± 5.3 ***	57.14 ± 4.1 ^b^	42.86 ± 4.1 ***

## Data Availability

Data are contained within the article and Supplementary Materials.

## Supplementary Materials

The following supporting information can be downloaded at: https://www.mdpi.com/article/10.3390/molecules30244679/s1.
