# Supplementary figures and images for: Natural Phytotherapeutics in Dermatology and Cosmetology: Bioactive Potential of Grape Pomace on Human Skin Fibroblasts
Source: Molecules. 2025 Dec 6;30(24):4679. doi: 10.3390/molecules30244679 (PMC12736252; doi:10.3390/molecules30244679)

## resveratrol

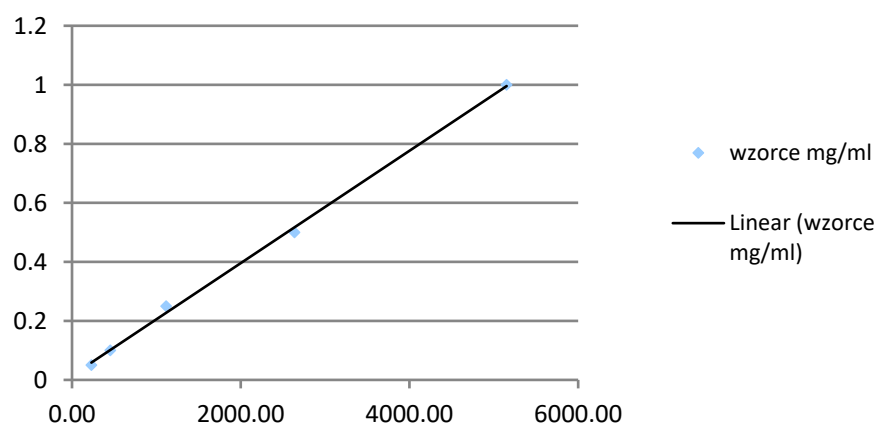

## verbascoside

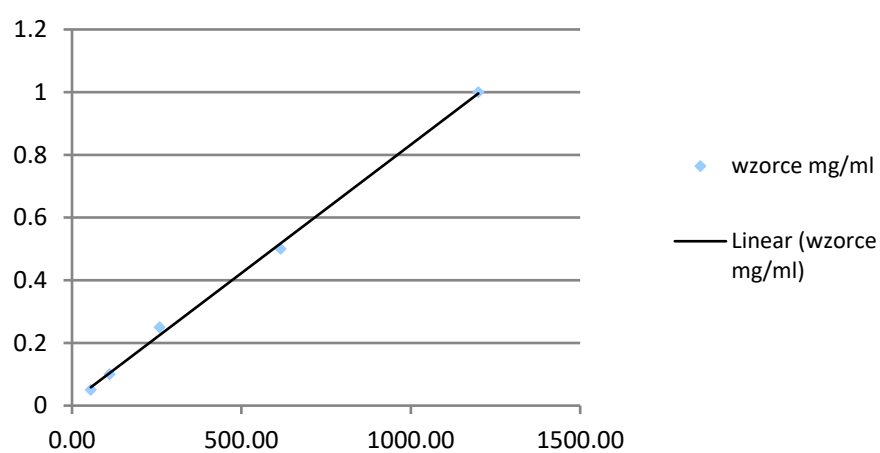

Supplement: Supplementary file 1 [file molecules-30-04679-s001.zip › HPLC calibration curves.pdf]

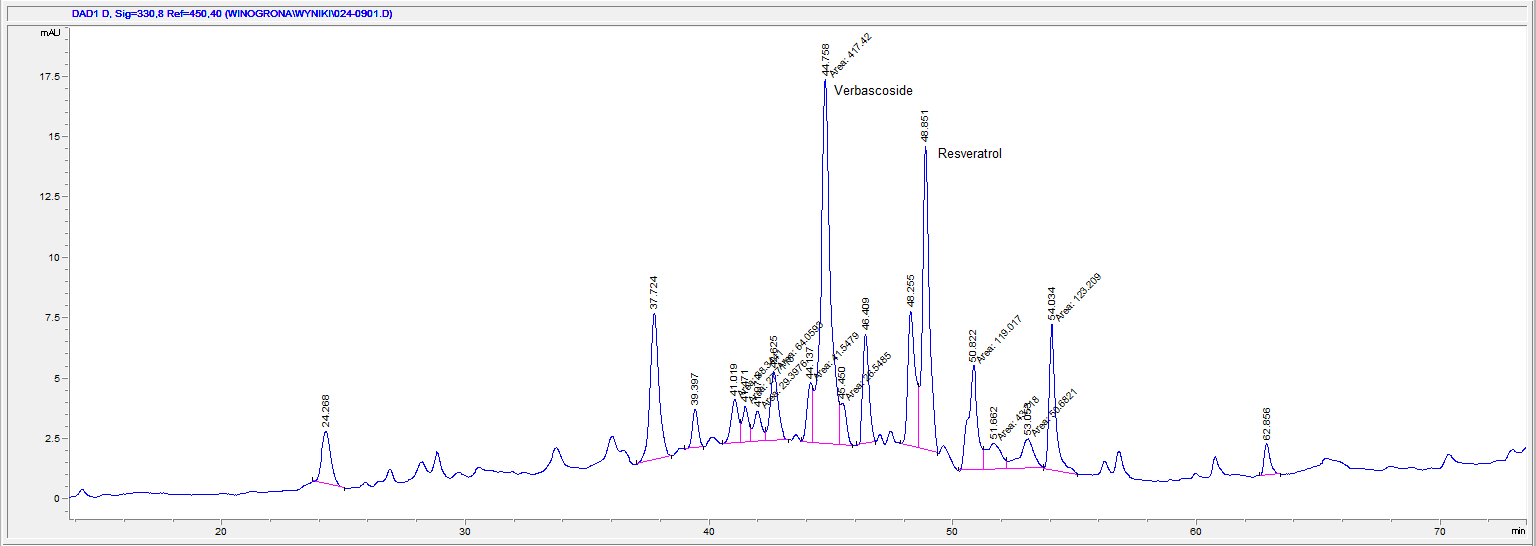

Supplement: Supplementary file 1 [file molecules-30-04679-s001.zip › Marechal Foch lyophilizate.png]

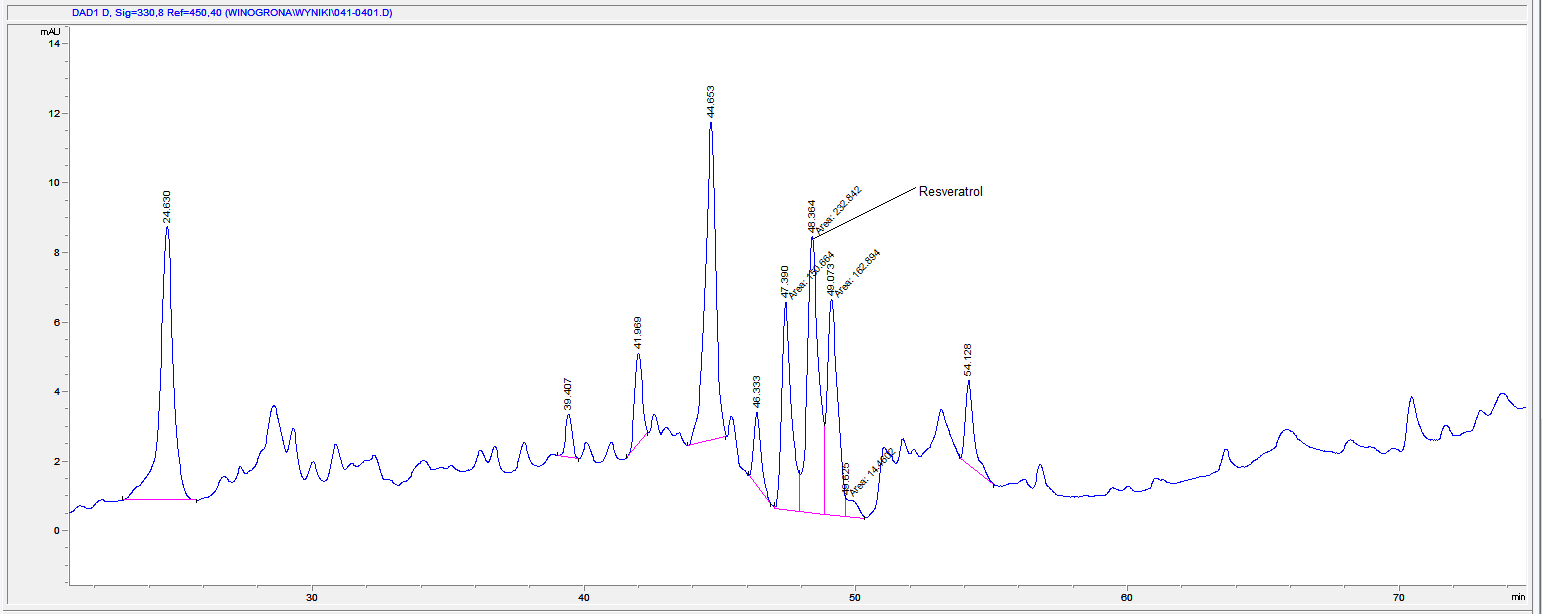

Supplement: Supplementary file 1 [file molecules-30-04679-s001.zip › marechal foch skins.png]

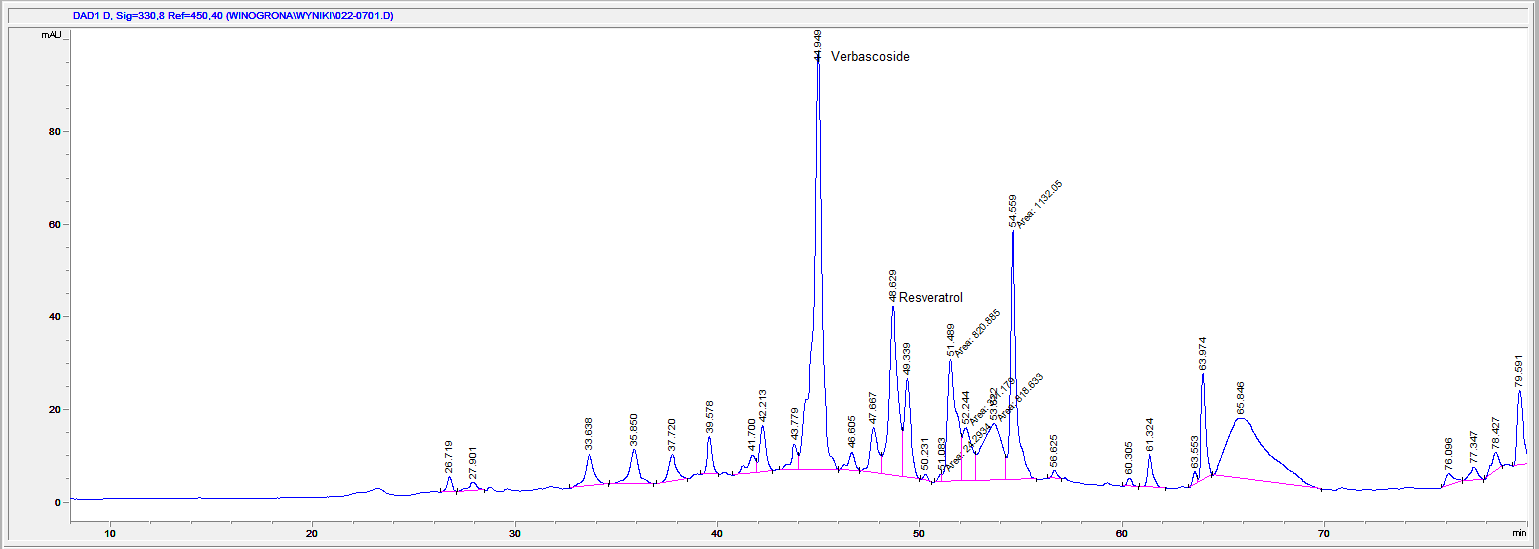

Supplement: Supplementary file 1 [file molecules-30-04679-s001.zip › Regent lyophilizate.png]

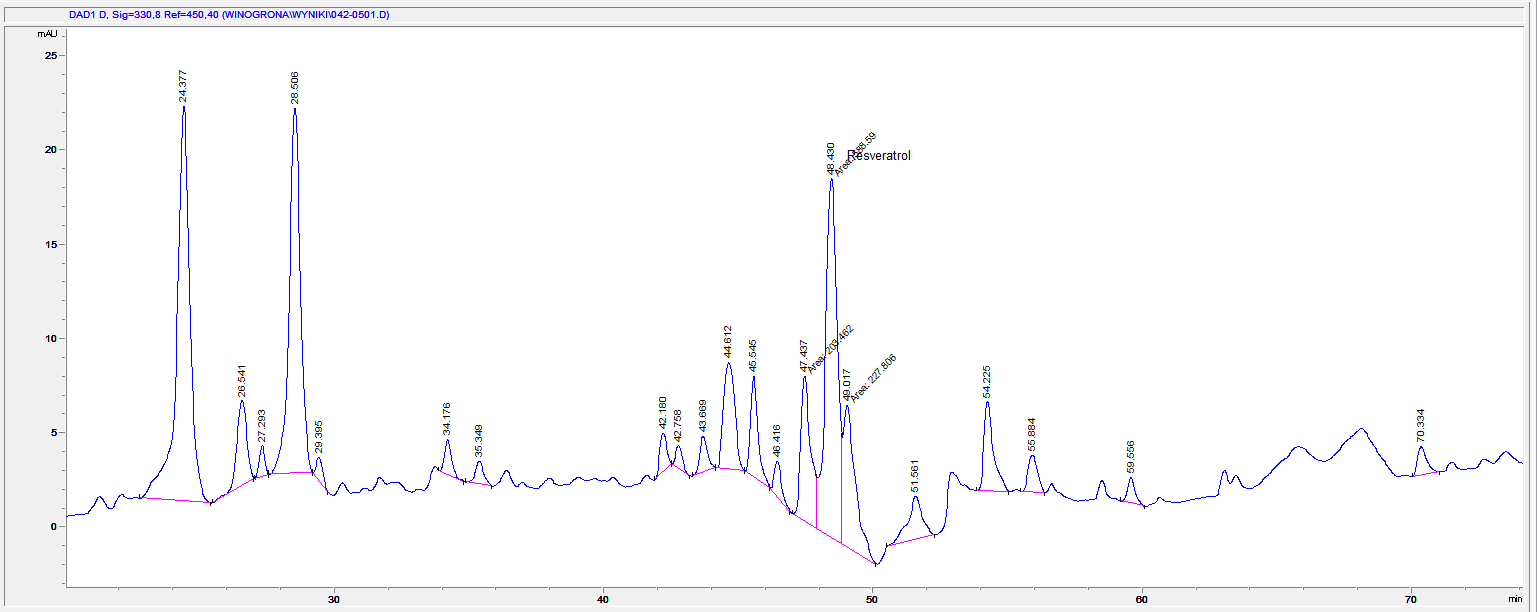

Supplement: Supplementary file 1 [file molecules-30-04679-s001.zip › Regent skins.png]

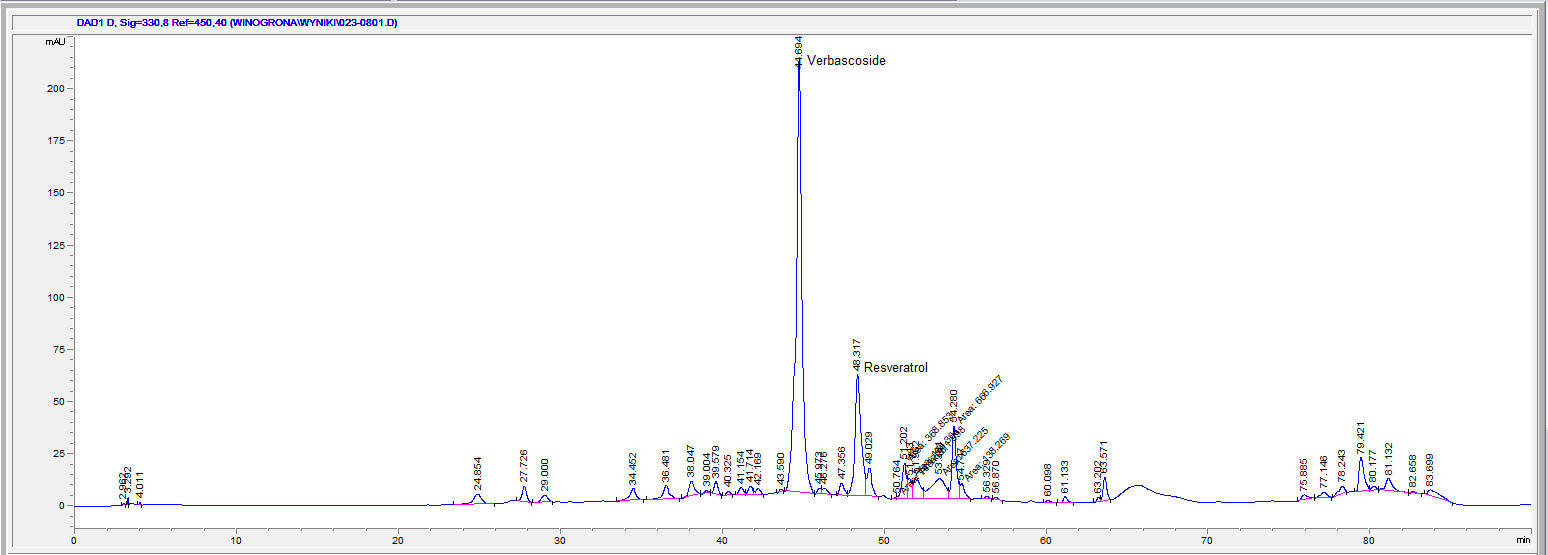

Supplement: Supplementary file 1 [file molecules-30-04679-s001.zip › Rondo lyophilizate.png]

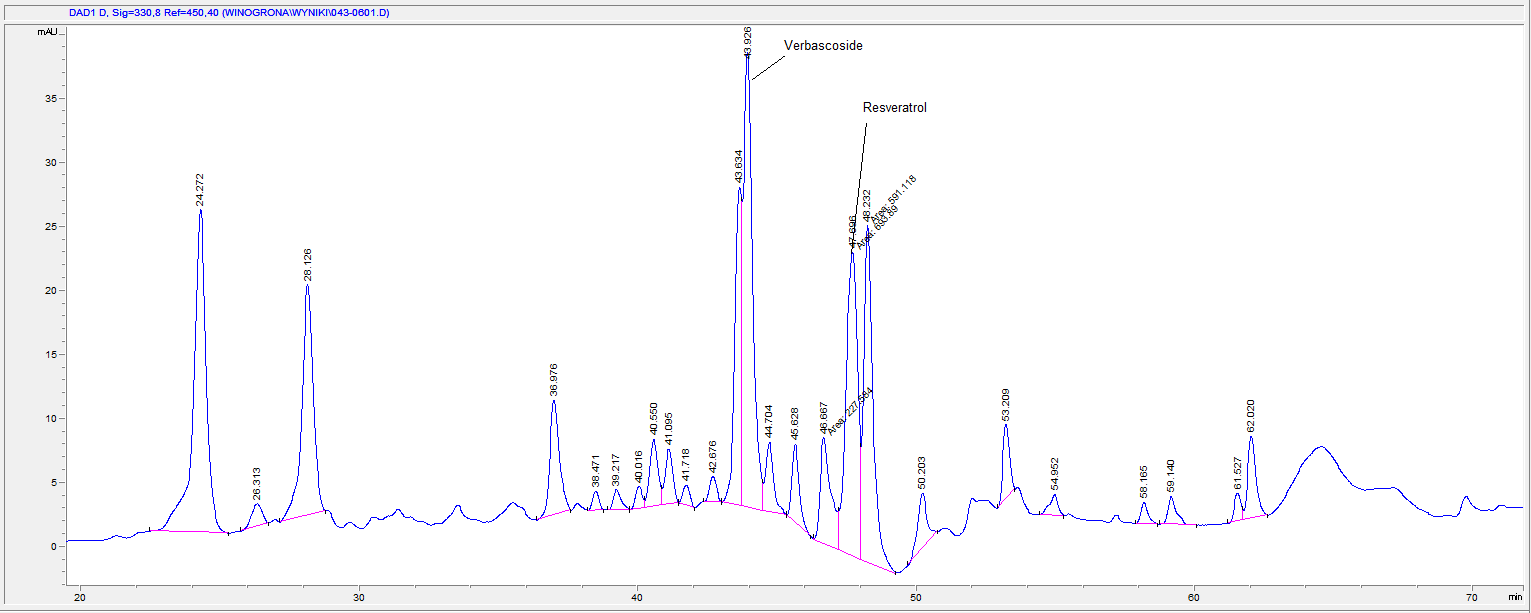

Supplement: Supplementary file 1 [file molecules-30-04679-s001.zip › Rondo skins.png]
